# Supplementary material for: Heparin-based hydrogel scaffolding alters the transcriptomic profile and increases the chemoresistance of MDA-MB-231 triple-negative breast cancer cells
Source: Biomater Sci. 2020 Feb 13;8(10):2786–96. doi: 10.1039/c9bm01481k (PMC7497406; doi:10.1039/c9bm01481k)
Supplement: Supplementary file 2 [file BM-008-C9BM01481K-s002.zip › Supplementary File 4/HP-B hydrogel + EGF (s)vControl/Pathways/my_analysis.Gsea.1545201170987/HALLMARK_OXIDATIVE_PHOSPHORYLATION.html]

Details for gene set HALLMARK\_OXIDATIVE\_PHOSPHORYLATION[GSEA]

|  || Dataset | expr.class.cls#HYDEGF\_versus\_CONTROL.class.cls#HYDEGF\_versus\_CONTROL\_repos |
| Phenotype | class.cls#HYDEGF\_versus\_CONTROL\_repos |
| Upregulated in class | CONTROL |
| GeneSet | HALLMARK\_OXIDATIVE\_PHOSPHORYLATION |
| Enrichment Score (ES) | -0.6035538 |
| Normalized Enrichment Score (NES) | -2.88618 |
| Nominal p-value | 0.0 |
| FDR q-value | 0.0 |
| FWER p-Value | 0.0 |
Table: GSEA Results Summary

  

Fig 1: Enrichment plot: HALLMARK\_OXIDATIVE\_PHOSPHORYLATION      
 Profile of the Running ES Score & Positions of GeneSet Members on the Rank Ordered List

  

| PROBE | DESCRIPTION (from dataset) | GENE SYMBOL | GENE\_TITLE | RANK IN GENE LIST | RANK METRIC SCORE | RUNNING ES | CORE ENRICHMENT || 1 | ACAA1 | na |  |  | 1222 | 1.561 | -0.0565 | No |
| 2 | NDUFA9 | na |  |  | 1642 | 1.379 | -0.0716 | No |
| 3 | ECI1 | na |  |  | 2082 | 1.242 | -0.0885 | No |
| 4 | CPT1A | na |  |  | 2216 | 1.207 | -0.0895 | No |
| 5 | MTRR | na |  |  | 2252 | 1.194 | -0.0854 | No |
| 6 | SURF1 | na |  |  | 2718 | 1.075 | -0.1044 | No |
| 7 | ATP6V0C | na |  |  | 3447 | 0.914 | -0.1382 | No |
| 8 | AFG3L2 | na |  |  | 3462 | 0.912 | -0.1343 | No |
| 9 | RHOT2 | na |  |  | 3705 | 0.863 | -0.1428 | No |
| 10 | PMPCA | na |  |  | 3732 | 0.858 | -0.1398 | No |
| 11 | BAX | na |  |  | 3746 | 0.856 | -0.1362 | No |
| 12 | TCIRG1 | na |  |  | 4465 | 0.739 | -0.1703 | No |
| 13 | ACADVL | na |  |  | 4708 | 0.706 | -0.1795 | No |
| 14 | COX10 | na |  |  | 4733 | 0.701 | -0.1773 | No |
| 15 | SLC25A12 | na |  |  | 4948 | 0.660 | -0.1852 | No |
| 16 | MRPL11 | na |  |  | 4953 | 0.659 | -0.1822 | No |
| 17 | NDUFV1 | na |  |  | 5074 | 0.642 | -0.1853 | No |
| 18 | POR | na |  |  | 5102 | 0.637 | -0.1835 | No |
| 19 | MFN2 | na |  |  | 6029 | 0.499 | -0.2297 | No |
| 20 | NDUFV2 | na |  |  | 6230 | 0.472 | -0.2379 | No |
| 21 | CYC1 | na |  |  | 6701 | 0.411 | -0.2606 | No |
| 22 | NDUFS7 | na |  |  | 6743 | 0.404 | -0.2607 | No |
| 23 | TIMM13 | na |  |  | 6850 | 0.390 | -0.2643 | No |
| 24 | TIMM50 | na |  |  | 7977 | 0.245 | -0.3223 | No |
| 25 | TIMM9 | na |  |  | 8204 | 0.223 | -0.3331 | No |
| 26 | HSD17B10 | na |  |  | 9087 | 0.117 | -0.3789 | No |
| 27 | TIMM8B | na |  |  | 9560 | 0.068 | -0.4034 | No |
| 28 | GOT2 | na |  |  | 9565 | 0.067 | -0.4033 | No |
| 29 | SLC25A20 | na |  |  | 9588 | 0.062 | -0.4042 | No |
| 30 | IDH3G | na |  |  | 9687 | 0.051 | -0.4091 | No |
| 31 | FDX1 | na |  |  | 9724 | 0.046 | -0.4107 | No |
| 32 | SDHA | na |  |  | 9749 | 0.043 | -0.4118 | No |
| 33 | AIFM1 | na |  |  | 10142 | -0.001 | -0.4324 | No |
| 34 | SLC25A11 | na |  |  | 10152 | -0.003 | -0.4328 | No |
| 35 | NDUFS8 | na |  |  | 10157 | -0.004 | -0.4330 | No |
| 36 | ETFB | na |  |  | 10598 | -0.050 | -0.4559 | No |
| 37 | NQO2 | na |  |  | 10723 | -0.068 | -0.4621 | No |
| 38 | SLC25A6 | na |  |  | 10805 | -0.078 | -0.4660 | No |
| 39 | PDHX | na |  |  | 10924 | -0.098 | -0.4717 | No |
| 40 | NDUFB2 | na |  |  | 11558 | -0.169 | -0.5042 | No |
| 41 | MRPS15 | na |  |  | 11712 | -0.188 | -0.5113 | No |
| 42 | FXN | na |  |  | 12066 | -0.228 | -0.5287 | No |
| 43 | ATP5D | na |  |  | 12070 | -0.228 | -0.5277 | No |
| 44 | LRPPRC | na |  |  | 12106 | -0.234 | -0.5284 | No |
| 45 | NNT | na |  |  | 12167 | -0.239 | -0.5304 | No |
| 46 | COX8A | na |  |  | 12211 | -0.246 | -0.5314 | No |
| 47 | OPA1 | na |  |  | 12251 | -0.253 | -0.5322 | No |
| 48 | SUPV3L1 | na |  |  | 12310 | -0.262 | -0.5339 | No |
| 49 | MRPS11 | na |  |  | 12329 | -0.266 | -0.5335 | No |
| 50 | GRPEL1 | na |  |  | 12492 | -0.285 | -0.5406 | No |
| 51 | MRPS12 | na |  |  | 12675 | -0.309 | -0.5487 | No |
| 52 | RETSAT | na |  |  | 12706 | -0.315 | -0.5487 | No |
| 53 | NDUFS3 | na |  |  | 12718 | -0.316 | -0.5477 | No |
| 54 | DLST | na |  |  | 12764 | -0.326 | -0.5484 | No |
| 55 | IMMT | na |  |  | 12982 | -0.338 | -0.5581 | No |
| 56 | ACO2 | na |  |  | 13111 | -0.355 | -0.5631 | No |
| 57 | HSPA9 | na |  |  | 13160 | -0.362 | -0.5638 | No |
| 58 | IDH3A | na |  |  | 13195 | -0.370 | -0.5637 | No |
| 59 | CS | na |  |  | 13254 | -0.378 | -0.5649 | No |
| 60 | MTRF1 | na |  |  | 13297 | -0.385 | -0.5652 | No |
| 61 | COX15 | na |  |  | 13341 | -0.393 | -0.5655 | No |
| 62 | GPI | na |  |  | 13512 | -0.415 | -0.5724 | No |
| 63 | IDH3B | na |  |  | 13523 | -0.417 | -0.5708 | No |
| 64 | ISCU | na |  |  | 13541 | -0.419 | -0.5696 | No |
| 65 | UQCRC1 | na |  |  | 13620 | -0.428 | -0.5716 | No |
| 66 | TOMM22 | na |  |  | 13666 | -0.436 | -0.5717 | No |
| 67 | MAOB | na |  |  | 13667 | -0.436 | -0.5696 | No |
| 68 | PDK4 | na |  |  | 13709 | -0.442 | -0.5695 | No |
| 69 | SDHD | na |  |  | 13743 | -0.448 | -0.5690 | No |
| 70 | COX11 | na |  |  | 13744 | -0.448 | -0.5668 | No |
| 71 | PHB2 | na |  |  | 13845 | -0.465 | -0.5697 | No |
| 72 | OXA1L | na |  |  | 13958 | -0.482 | -0.5732 | No |
| 73 | IDH2 | na |  |  | 14028 | -0.492 | -0.5743 | No |
| 74 | ECH1 | na |  |  | 14216 | -0.507 | -0.5816 | No |
| 75 | VDAC3 | na |  |  | 14280 | -0.516 | -0.5824 | No |
| 76 | SUCLA2 | na |  |  | 14341 | -0.527 | -0.5829 | No |
| 77 | ECHS1 | na |  |  | 14355 | -0.530 | -0.5809 | No |
| 78 | GPX4 | na |  |  | 14401 | -0.538 | -0.5806 | No |
| 79 | CYB5R3 | na |  |  | 14411 | -0.540 | -0.5784 | No |
| 80 | MDH1 | na |  |  | 14491 | -0.555 | -0.5798 | No |
| 81 | ATP6V1H | na |  |  | 14532 | -0.560 | -0.5791 | No |
| 82 | ACADM | na |  |  | 14777 | -0.601 | -0.5889 | No |
| 83 | TIMM17A | na |  |  | 14957 | -0.628 | -0.5952 | No |
| 84 | SLC25A5 | na |  |  | 14995 | -0.632 | -0.5940 | No |
| 85 | SLC25A3 | na |  |  | 15128 | -0.659 | -0.5976 | No |
| 86 | LDHB | na |  |  | 15225 | -0.674 | -0.5993 | No |
| 87 | ATP5G1 | na |  |  | 15307 | -0.688 | -0.6001 | Yes |
| 88 | ACAT1 | na |  |  | 15338 | -0.694 | -0.5982 | Yes |
| 89 | MRPL35 | na |  |  | 15399 | -0.701 | -0.5979 | Yes |
| 90 | ATP5A1 | na |  |  | 15456 | -0.713 | -0.5972 | Yes |
| 91 | OGDH | na |  |  | 15464 | -0.715 | -0.5940 | Yes |
| 92 | NDUFC2 | na |  |  | 15530 | -0.727 | -0.5938 | Yes |
| 93 | UQCRC2 | na |  |  | 15532 | -0.728 | -0.5902 | Yes |
| 94 | NDUFB5 | na |  |  | 15542 | -0.731 | -0.5870 | Yes |
| 95 | ISCA1 | na |  |  | 15576 | -0.738 | -0.5851 | Yes |
| 96 | CYCS | na |  |  | 15592 | -0.741 | -0.5822 | Yes |
| 97 | UQCR11 | na |  |  | 15617 | -0.747 | -0.5797 | Yes |
| 98 | MDH2 | na |  |  | 15652 | -0.754 | -0.5777 | Yes |
| 99 | ATP6V1C1 | na |  |  | 15672 | -0.757 | -0.5749 | Yes |
| 100 | CASP7 | na |  |  | 15674 | -0.757 | -0.5712 | Yes |
| 101 | RHOT1 | na |  |  | 15746 | -0.773 | -0.5711 | Yes |
| 102 | MRPS22 | na |  |  | 15883 | -0.798 | -0.5742 | Yes |
| 103 | PDHA1 | na |  |  | 15909 | -0.803 | -0.5715 | Yes |
| 104 | HADHA | na |  |  | 16033 | -0.828 | -0.5738 | Yes |
| 105 | SLC25A4 | na |  |  | 16080 | -0.840 | -0.5721 | Yes |
| 106 | FH | na |  |  | 16089 | -0.841 | -0.5683 | Yes |
| 107 | ALAS1 | na |  |  | 16248 | -0.886 | -0.5722 | Yes |
| 108 | TIMM10 | na |  |  | 16261 | -0.889 | -0.5683 | Yes |
| 109 | DLD | na |  |  | 16317 | -0.903 | -0.5667 | Yes |
| 110 | CYB5A | na |  |  | 16333 | -0.907 | -0.5630 | Yes |
| 111 | MRPL15 | na |  |  | 16371 | -0.917 | -0.5603 | Yes |
| 112 | ABCB7 | na |  |  | 16402 | -0.924 | -0.5573 | Yes |
| 113 | UQCRFS1 | na |  |  | 16481 | -0.942 | -0.5567 | Yes |
| 114 | ATP6V0B | na |  |  | 16582 | -0.965 | -0.5571 | Yes |
| 115 | ATP6V1D | na |  |  | 16591 | -0.967 | -0.5527 | Yes |
| 116 | DECR1 | na |  |  | 16731 | -1.006 | -0.5550 | Yes |
| 117 | NDUFB7 | na |  |  | 16774 | -1.017 | -0.5521 | Yes |
| 118 | NDUFC1 | na |  |  | 16787 | -1.023 | -0.5476 | Yes |
| 119 | HTRA2 | na |  |  | 16814 | -1.031 | -0.5438 | Yes |
| 120 | GLUD1 | na |  |  | 16838 | -1.039 | -0.5398 | Yes |
| 121 | VDAC2 | na |  |  | 16846 | -1.042 | -0.5350 | Yes |
| 122 | HADHB | na |  |  | 16850 | -1.042 | -0.5299 | Yes |
| 123 | NDUFS2 | na |  |  | 16865 | -1.045 | -0.5254 | Yes |
| 124 | NDUFS6 | na |  |  | 16937 | -1.066 | -0.5238 | Yes |
| 125 | ETFDH | na |  |  | 16947 | -1.071 | -0.5190 | Yes |
| 126 | ATP5B | na |  |  | 16949 | -1.072 | -0.5136 | Yes |
| 127 | ETFA | na |  |  | 16990 | -1.086 | -0.5103 | Yes |
| 128 | HCCS | na |  |  | 17006 | -1.091 | -0.5056 | Yes |
| 129 | COX7A2L | na |  |  | 17201 | -1.144 | -0.5101 | Yes |
| 130 | NDUFS1 | na |  |  | 17222 | -1.152 | -0.5054 | Yes |
| 131 | ATP6V0E1 | na |  |  | 17278 | -1.173 | -0.5024 | Yes |
| 132 | NDUFA3 | na |  |  | 17285 | -1.175 | -0.4969 | Yes |
| 133 | SDHB | na |  |  | 17291 | -1.178 | -0.4912 | Yes |
| 134 | NDUFA5 | na |  |  | 17332 | -1.194 | -0.4874 | Yes |
| 135 | ATP6AP1 | na |  |  | 17435 | -1.228 | -0.4866 | Yes |
| 136 | PRDX3 | na |  |  | 17543 | -1.275 | -0.4858 | Yes |
| 137 | VDAC1 | na |  |  | 17636 | -1.308 | -0.4841 | Yes |
| 138 | MTX2 | na |  |  | 17659 | -1.320 | -0.4787 | Yes |
| 139 | ATP5O | na |  |  | 17678 | -1.320 | -0.4730 | Yes |
| 140 | NDUFB3 | na |  |  | 17730 | -1.349 | -0.4690 | Yes |
| 141 | ATP6V1G1 | na |  |  | 17762 | -1.364 | -0.4638 | Yes |
| 142 | ATP6V1F | na |  |  | 17866 | -1.424 | -0.4621 | Yes |
| 143 | UQCRQ | na |  |  | 17923 | -1.453 | -0.4578 | Yes |
| 144 | MRPL34 | na |  |  | 17933 | -1.459 | -0.4509 | Yes |
| 145 | ATP5F1 | na |  |  | 17963 | -1.470 | -0.4451 | Yes |
| 146 | PDP1 | na |  |  | 17969 | -1.472 | -0.4380 | Yes |
| 147 | DLAT | na |  |  | 17988 | -1.481 | -0.4315 | Yes |
| 148 | ACAA2 | na |  |  | 18083 | -1.534 | -0.4288 | Yes |
| 149 | UQCR10 | na |  |  | 18089 | -1.539 | -0.4214 | Yes |
| 150 | ALDH6A1 | na |  |  | 18145 | -1.576 | -0.4164 | Yes |
| 151 | SDHC | na |  |  | 18153 | -1.583 | -0.4088 | Yes |
| 152 | SUCLG1 | na |  |  | 18207 | -1.604 | -0.4036 | Yes |
| 153 | ACADSB | na |  |  | 18229 | -1.620 | -0.3966 | Yes |
| 154 | ATP1B1 | na |  |  | 18250 | -1.638 | -0.3895 | Yes |
| 155 | NDUFB6 | na |  |  | 18260 | -1.645 | -0.3817 | Yes |
| 156 | ATP5C1 | na |  |  | 18271 | -1.654 | -0.3739 | Yes |
| 157 | UQCRH | na |  |  | 18349 | -1.711 | -0.3694 | Yes |
| 158 | ATP5H | na |  |  | 18402 | -1.757 | -0.3634 | Yes |
| 159 | OAT | na |  |  | 18403 | -1.757 | -0.3546 | Yes |
| 160 | MRPS30 | na |  |  | 18417 | -1.769 | -0.3464 | Yes |
| 161 | COX7A2 | na |  |  | 18424 | -1.774 | -0.3379 | Yes |
| 162 | MGST3 | na |  |  | 18477 | -1.823 | -0.3315 | Yes |
| 163 | UQCRB | na |  |  | 18494 | -1.832 | -0.3232 | Yes |
| 164 | COX17 | na |  |  | 18505 | -1.841 | -0.3145 | Yes |
| 165 | NDUFA6 | na |  |  | 18511 | -1.845 | -0.3055 | Yes |
| 166 | BDH2 | na |  |  | 18527 | -1.857 | -0.2970 | Yes |
| 167 | NDUFB1 | na |  |  | 18573 | -1.893 | -0.2899 | Yes |
| 168 | NDUFAB1 | na |  |  | 18589 | -1.910 | -0.2811 | Yes |
| 169 | NDUFA2 | na |  |  | 18604 | -1.930 | -0.2722 | Yes |
| 170 | NDUFB4 | na |  |  | 18632 | -1.965 | -0.2638 | Yes |
| 171 | ATP5L | na |  |  | 18649 | -1.991 | -0.2547 | Yes |
| 172 | COX4I1 | na |  |  | 18654 | -1.993 | -0.2449 | Yes |
| 173 | ATP5G2 | na |  |  | 18689 | -2.035 | -0.2365 | Yes |
| 174 | IDH1 | na |  |  | 18690 | -2.035 | -0.2263 | Yes |
| 175 | ATP5J | na |  |  | 18717 | -2.073 | -0.2173 | Yes |
| 176 | NDUFS4 | na |  |  | 18740 | -2.102 | -0.2080 | Yes |
| 177 | PDHB | na |  |  | 18748 | -2.110 | -0.1978 | Yes |
| 178 | PHYH | na |  |  | 18766 | -2.139 | -0.1880 | Yes |
| 179 | ATP5G3 | na |  |  | 18792 | -2.177 | -0.1784 | Yes |
| 180 | NDUFA4 | na |  |  | 18804 | -2.198 | -0.1680 | Yes |
| 181 | COX5A | na |  |  | 18911 | -2.373 | -0.1617 | Yes |
| 182 | ATP5I | na |  |  | 18920 | -2.397 | -0.1501 | Yes |
| 183 | NDUFB8 | na |  |  | 18941 | -2.432 | -0.1390 | Yes |
| 184 | ATP6V1E1 | na |  |  | 18949 | -2.457 | -0.1271 | Yes |
| 185 | ATP5J2 | na |  |  | 18983 | -2.543 | -0.1161 | Yes |
| 186 | COX5B | na |  |  | 18986 | -2.552 | -0.1034 | Yes |
| 187 | COX6B1 | na |  |  | 19007 | -2.599 | -0.0915 | Yes |
| 188 | ATP5E | na |  |  | 19028 | -2.665 | -0.0792 | Yes |
| 189 | COX7B | na |  |  | 19032 | -2.677 | -0.0660 | Yes |
| 190 | NDUFA1 | na |  |  | 19075 | -2.807 | -0.0541 | Yes |
| 191 | COX6A1 | na |  |  | 19097 | -2.897 | -0.0408 | Yes |
| 192 | COX6C | na |  |  | 19125 | -3.037 | -0.0270 | Yes |
| 193 | NDUFA8 | na |  |  | 19127 | -3.044 | -0.0118 | Yes |
| 194 | COX7C | na |  |  | 19132 | -3.095 | 0.0035 | Yes |
Table: GSEA details [plain text format]

  

Fig 2: HALLMARK\_OXIDATIVE\_PHOSPHORYLATION      
 Blue-Pink O' Gram in the Space of the Analyzed GeneSet

  

Fig 3: HALLMARK\_OXIDATIVE\_PHOSPHORYLATION: Random ES distribution      
 Gene set null distribution of ES for **HALLMARK\_OXIDATIVE\_PHOSPHORYLATION**
